# Critical Analysis on Characterization, Systemic Effect, and Therapeutic Potential of Beta-Sitosterol: A Plant-Derived Orphan Phytosterol

**DOI:** 10.3390/medicines3040029

**Published:** 2016-11-15

**Authors:** Muhammad Shahdaat Bin Sayeed, Selim Muhammad Rezaul Karim, Tasnuva Sharmin, Mohammed Monzur Morshed

**Affiliations:** 1 Department of Clinical Pharmacy and Pharmacology, University of Dhaka, Dhaka-1000, Bangladesh; 2 Department of Pharmacy, Daffodil International University, Dhaka-1207, Bangladesh; smroney46@gmail.com; 3 Department of Pharmaceutical Chemistry, University of Dhaka, Dhaka-1000, Bangladesh; tasnuva.phr.du@gmail.com; 4 Department of Biochemistry and Molecular, Biology, University of Dhaka, Dhaka-1000, Bangladesh; monzurmorshed.du@gmail.com

**Keywords:** beta-sitosterol, orphan phytosterol, characterization, therapeutic potential, drug delivery

## Abstract

Beta-sitosterol (BS) is a phytosterol, widely distributed throughout the plant kingdom and known to be involved in the stabilization of cell membranes. To compile the sources, physical and chemical properties, spectral and chromatographic analytical methods, synthesis, systemic effects, pharmacokinetics, therapeutic potentials, toxicity, drug delivery and finally, to suggest future research with BS, classical as well as on-line literature were studied. Classical literature includes classical books on ethnomedicine and phytochemistry, and the electronic search included Pubmed, SciFinder, Scopus, the Web of Science, Google Scholar, and others. BS could be obtained from different plants, but the total biosynthetic pathway, as well as its exact physiological and structural function in plants, have not been fully understood. Different pharmacological effects have been studied, but most of the mechanisms of action have not been studied in detail. Clinical trials with BS have shown beneficial effects in different diseases, but long-term study results are not available. These have contributed to its current status as an “orphan phytosterol”. Therefore, extensive research regarding its effect at cellular and molecular level in humans as well as addressing the claims made by commercial manufacturers such as the cholesterol lowering ability, immunological activity etc. are highly recommended.

## 1. Introduction

Beta-sitosterol (BS) is one of the several phytosterols with a chemical structure similar to that of cholesterol [1]. It is a natural micronutrient in higher plants and is found in the serum and tissues of healthy individuals at a concentration 800–1000 times lower than that of endogenous cholesterol. Its glycoside, sitosterolin, is also present in serum, but in lower concentration [2]. These molecules are synthesized in plants; whereas animals obtain them through diet [1].

The Joint FAO/WHO Expert Committee on Food Additives (JECFA) published scientific opinions on phytosterols without specific directives on BS separately [3]. In a series of scientific publications by the European Food Safety Authority (EFSA), BS was also not mentioned singly [4]. BS is generally considered as a safe, natural, and effective nutritional supplement and has been shown to have many potential benefits. Administration of BS in rats is found not to cause genotoxicity and cytotoxicity [5]. BS possesses antioxidant, antimicrobial, angiogenic, antioxidant, immunomodulatory, antidiabetic, anti-inflammatory, anticancer, and antinociceptive activities without major toxicity. There are some nutraceutical preparations available on the market which contain BS. Their manufacturers claim many beneficial effects without substantial experimental evidence. The recent pace of research with BS has been slowed down significantly and has left this molecule as an “orphan phytosterol” [1,6,7,8].

There are some reviews regarding the effects of phytosterols on health [9,10,11] and some reviews on the beneficial effects of phytosterol on a specific disease [12], but there is no single review regarding critical analysis of the current knowledge, the gaps in the knowledge on BS, and the necessity of future research to be conducted with BS to fill those gaps. The purpose of this review is to describe the known sources, characteristics, biosynthesis, chemical synthesis, pharmacological and toxicological effects of BS in order to emphasize its significance as well as the limitation of information on BS in order to set an avenue for further study with BS.

## 2. Sources

BS has been reported to be present in various dietary and non-dietary plants [13]. It exists in different plant parts such as leaves [14], rhizomes [15], and fruits [16]. It has also been reported to be present in different plant tissue cultures [17]. Studies have been reported regarding its membrane stabilizing effect on cell membrane [18], but its role in the cytoplasm and chloroplast has not been studied yet [19,20]. BS-derived phytoecdysteroid is higher in plant tissues which are the most important chemical substance for plant survival, but whether or not BS has a significant role in plant protection needs further research [21].

## 3. Characterization

### 3.1. Physical and Chemical Properties

**Figure 1:**
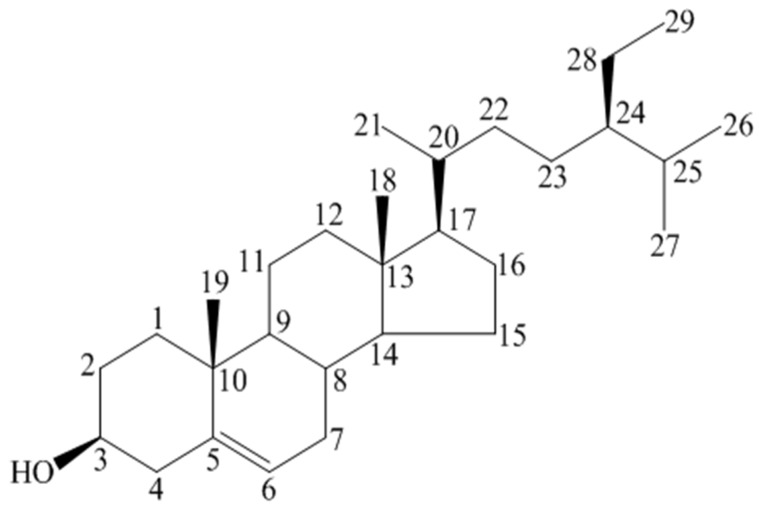
Beta-sitosterol(BS) [3] (Drawn by using *ChemDraw* software).

BS (Figure 1) is a white, waxy powder with a characteristic odor. Its molecular formula is C_29_H_50_O, melting point is 139–142 °C and PubChem CID is 222284 (PubChem, 2015). It is thermally unstable and converted to oxidized products [22].

**Figure 2:**
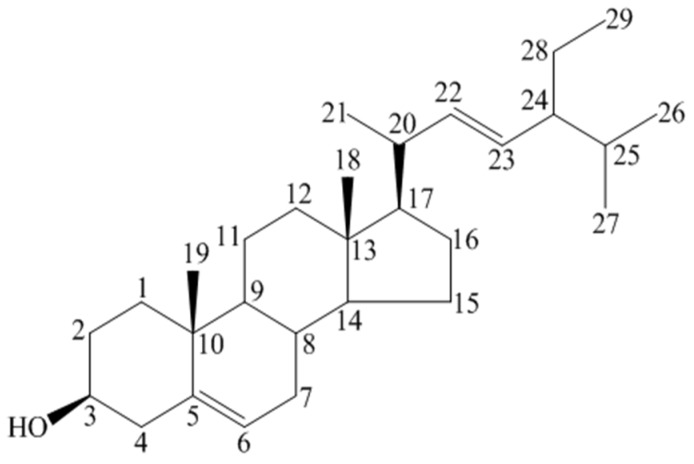
Stigmasterol [3] (Drawn by using *ChemDraw* software).

**Figure 3:**
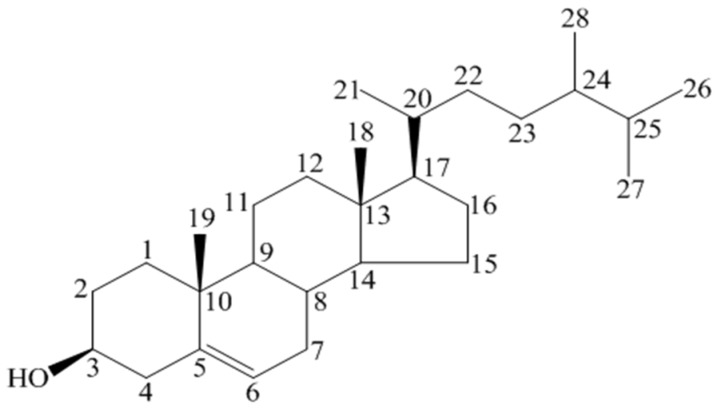
Campesterol [3] (Drawn by using *ChemDraw* software).

Although there is information on the oxidized products, only limited information regarding the physiological, pharmacological and pathological effects of those products is available [23,24]. It is hydrophobic and soluble in alcohols but has been observed to exist in three different forms based on the number of water molecules added: anhydrous, hemihydrate, and monohydrate. Monohydrate BS forms needle-shaped crystals instead of plate-like anhydrous crystals and structured suspensions with shear thinning behavior [25]. Its IUPAC name is 17-(5-Ethyl-6-methyl heptane-2-yl)-10,13-dimethyl-2,3,4,7,8,9,11,12,14,15,16,17-dodecahydro-1H-cyclopenta[a]phenanthren-3-ol. BS is also mentioned as β-sitosterol, (3β)-stigmast-5-en-3-ol, 22:23-dihydro stigmasterol, α-dihydro fucosterol, cinchol, cupreol, rhamnol, quebrachol, and sitosterin. Some other most prevalent plant sterols are campesterol (methyl group at C24), sitosterol (ethyl group at C24), brassicasterol (methyl group at C24, Δ22), and stigmasterol (ethyl group at C24, Δ22) [26]. The molecular formula of Stigmasterol is C_29_H_48_O (Figure 2), its melting point is 170 °C, and the PubChem CID is 5280794. For Campesterol, the molecular formula is C_28_H_48_O (Figure 3) and the PubChem CID is 173183 (PubChem, 2015).

### 3.2. Spectral Analysis

For the detection of BS, various methods have been developed to analyze singly or simultaneously with food [27], vegetable oil [28], plasma [29,30], and dosage form [31].

#### 3.2.1. IR Spectral Analysis

The IR spectral analysis reveals a broad peak at 3549.99 cm^−1^ for the OH group, 2935.73 cm^−1^ for the CH2 group, 2867.38 cm^−1^ for CH group, 1637.63 cm^−1^ for the C=C group, and 1063.34 cm^−1^ for the C–O group. The molecular weight determination indicates C_29_H_50_O as its molecular formula [32]. Similar results were observed in which IR peaks were obtained at 3426.89, 2924.52, 2855.1, 1738.51, and 1057.31 cm^−1^ [33].

#### 3.2.2. NMR Spectral Analysis

The ^1^H NMR spectrum showed the presence of an olefinic signal (δH 5.08), indicating a >C=C< system in the ring. A one proton broad multiplet at δH 4.44 showed a cross peak with C-2 protons and a C-4 proton in HETCOR and this signal was assigned to a C-3 methine proton. A plethora of multiplets was found in the range δH 1.1–2.14 which was informative in the presence of different methylene and methine protons of the steroidal structure. The other proton resonances were allocated to the glucopyranoside. Further evidence was also provided by the ^13^C NMR (detail is provided in ref. [34]) that showed resonances for [35] carbon atoms.

The C3 carbon resonated at 71.73 ppm. The anomeric and the oxygenated methylene carbons of the sugar appeared at 100 and 61 ppm respectively. Thus on the basis of the spectral data, the structure of the compound was elucidated as stigmast-5-en-3-*O*-β-d-glucopyranoside (β sitosterol glucoside) [34].

Another study showed that the ^1^H NMR spectrum (400 MHz, CDCl_3_) of BS (Table 1) shows a proton corresponding to the proton connected to the C-3 hydroxyl group which appears as a triplet of a doublet of doublets at δ 3.53, the position and multiplicity of which is indicative of the steroid nucleus. The typical olefinic H-6 of the steroid skeleton is evident as a triplet (*J* = 6.4) at δ 5.36 that integrates for one proton. The spectrum further reveals two singlets at δ 0.68 and 1.01 ppm each of three proton intensity, assigned to two tertiary methyl groups at C-18 and C-19, respectively. The three proton intensity, is assigned to two tertiary methyl groups at C-18 and C-19, respectively. The NMR spectrum also displays two doublets (*J* = 6.4) at δ 0.83 and 0.81 which is attributable to two methyl groups at C-26 and C-27. The doublets (*J* = 6.5) at δ 0.93 are ascribed to a methyl group at C-21. On the other hand, the triplet at δ 0.84 (*J* = 7.2) of three proton intensity is assigned to the primary methyl group attached to C-29 [35].

The ^13^C NMR together with COSY, HMQC, and HMBC shows twenty-nine carbon signals including six methyls, eleven methylenes, ten methane, and three quaternary carbons [35].

### 3.3. Chromatographic Analysis

#### 3.3.1. Thin Layer Chromatography (TLC)

In Thin layer chromatography (TLC) analysis of BS revealed a *R*_f_ value of 0.55 when the crystals were reconstituted in chloroform and spotted on the TLC plate in *n*-hexane: acetone (80:20) mobile phase system [32].

#### 3.3.2. Gas Layer Chromatography

To analyze BS, a Gas-liquid chromatography (GLC) method using the butyl ester of BS was implemented [27]. It was evaluated and found that an immobile phase of 1% SE-30 coated on 100–120 mesh Gas-Chrom Q packed in a 6 × 4 mm id glass column operated at 255 °C is the most efficient column.

#### 3.3.3. High-Performance Liquid Chromatography

Different High-Performance Liquid Chromatography (HPLC) methods have been developed for analyzing BS such as a narrow-bore HPLC-UV method [28]; a high-performance thin-layer chromatography densitometric method [28]; an HPLC method for qualitative analysis [36]; an HPLC method with online evaporative light scattering detector (ELSD) [31]; an HPLC/MS method using solvent combination of water/methanol vs. methanol/acetone/n-hexane applied on a Purospher Star RP-18e (125 × 2 mm, 3 micron) column [29].

#### 3.3.4. Gas Chromatography Mass Spectrometry (GCMS)

Ahmida et al. (2006) developed capillary gas chromatography coupled to mass spectrometry (GC-MS) for simultaneous detection of BS from other sterols by multiple selected ion monitoring. This method is based on the alkaline hydrolysis of sterol esters, extraction of free sterols and derivatization. The recovery of all sterols was in the range 76%–101% [30]. Most of these techniques are laborious and time-consuming [23] except for a recent method described by Srividya et al. (2014) [37]. Alkaline hydrolysis and liquid–liquid extraction followed by parallel detection on GC-FID and GC-MS is proposed as an ideal methodology for the bio-analysis of phytosterols [38]. Therefore, there is plenty of scopes to improve the efficiency as well as the limit of detection and quantification.

## 4. Synthesis

### 4.1. Biosynthesis

The exact biosynthetic mechanism of BS varies according to organisms, but generally, it follows the mevalonate pathway [39]. BS is biologically synthesized from both mevalonate and deoxyxylulose pathways [20] but prioritizes both or one of the pathways based on the external environment. Using ^13^C-labeling approach, the mechanism of BS biosynthesis was studied and it was proposed that isopentenyl diphosphate (IPP) combines with dimethylallyl diphosphate (DMAPP) to form farnesyl diphosphate (FPP) and then two molecules of FPP combine tail-to-tail to form Squalene, a triterpene and then cycloartenol which eventually forms BS by methylation, hydride shift, reduction, and slight modification in the beta-ring [19].

### 4.2. Comparison of Biosynthesis of BS and Cholesterol

**Figure 4:**
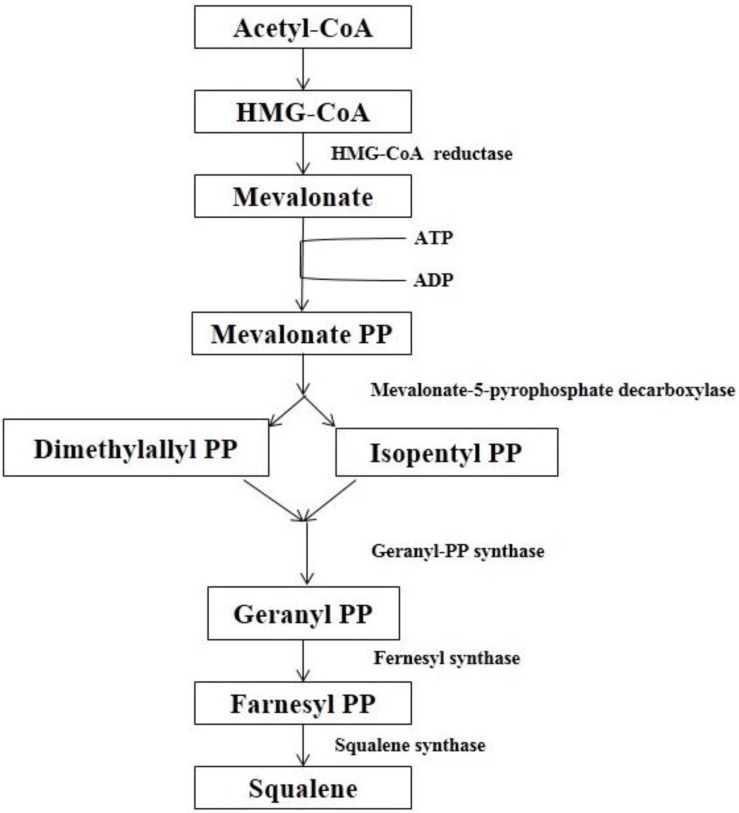
Formation of squalene [42].

**Figure 5:**
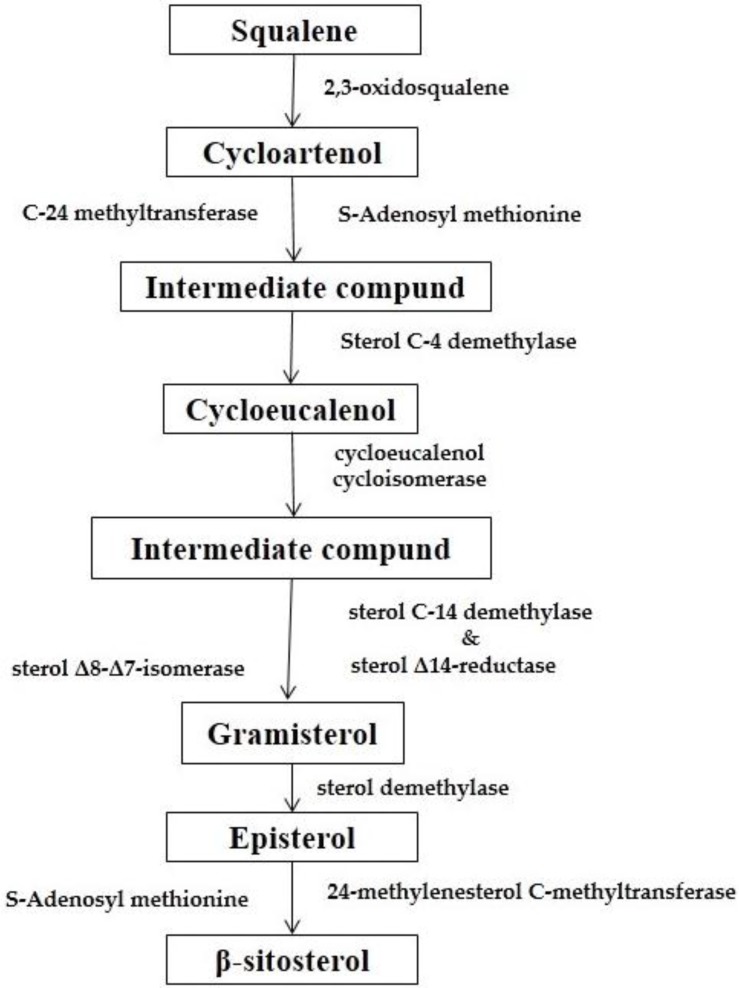
Synthesis of BS [40].

Both the biosynthesis of BS and Cholesterol follow the same direction till the formation of Squalene (Figure 4). However, the fate of Squalene varies due to the different target product. In the case of the biosynthesis of BS, Squalene forms cycloartenol through a cyclization reaction with 2,3-oxidosqualene. The double bond of cycloartenol is methylated by *S*-Adenosyl methionine (SAM) to give a carbocation that undergoes a hydride shift and loses a proton to yield an intermediate compound with a methylene side-chain. Both of these steps are catalyzed by sterol C-24 methyltransferase. The intermediate compound is then catalyzed by sterol C-4 demethylase and loses a methyl group to produce cyclo-eucalenol. Subsequent to this, the cyclopropane ring is opened with cyclo-eucalenol cyclo-isomerase to form another intermediate compound. This intermediate compound then loses a methyl group and undergoes an allylic isomerization to form gramisterol. This step is catalyzed by sterol C-14 demethylase, sterol Δ14-reductase, and sterol Δ8-Δ7-isomerase. The last methyl group is removed by sterol demethylase to form episterol. Finally, episterol is converted to β-sitosterol through methylation by SAM, reduction by NADPH, and modifications in the β-ring. Here 24-methylenesterol *C*-methyltransferase plays a very important role [40] (Figure 5).

**Figure 6:**
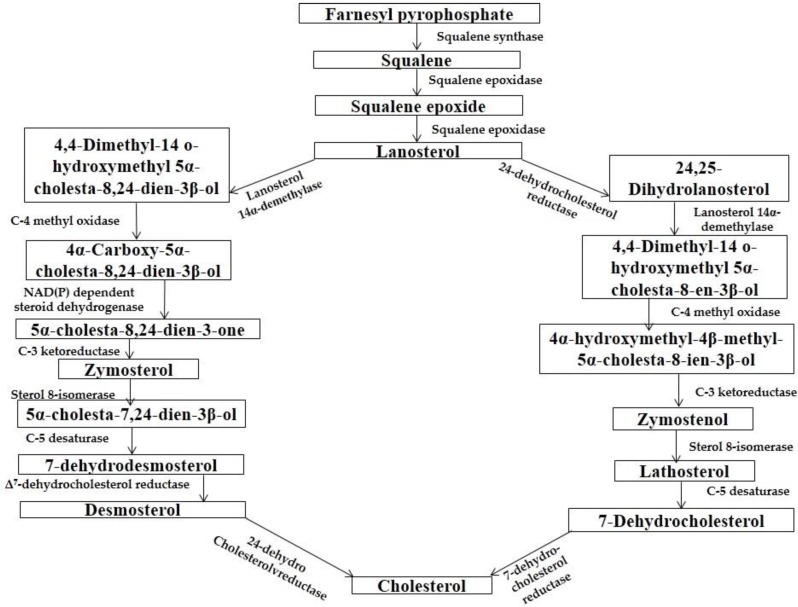
Synthesis of cholesterol [41].

The synthesis of cholesterol occurs in three stages, with the first stage taking place in the cytoplasm and the second and third stages occurring in the endoplasmic reticulum. The stages are (1) Synthesis of isopentenyl pyrophosphate, the “building block” of cholesterol; (2) Formation of squalene via the condensation of six molecules of isopentenyl phosphate and; (3) Conversion of squalene to cholesterol via several enzymatic reactions [41] (Figure 6).

### 4.3. Chemical Synthesis

Different routes have been reported for the synthesis of BS over more than 50 years. The selective hydrogenation of the stigmasterol side chain Δ^20−21^ alkene was found to produce BS contaminated with varying amounts of recovered stigmasterol as well as the fully saturated stigmastanol [43,44]. In another approach, the synthesis of sitosterol and related sterols circumvents the need for selective hydrogenation for protecting the Δ^5−6^ alkene as cyclopropyl carbinyl ether. Following hydrogenation of the Δ^22−23^ double bond, solvolysis of the cyclopropane reintroduces both the C3-alcohol and the Δ^5−6^ alkene [45,46]. Recently a new strategy for the synthesis of the side chain has modified phytosterols based upon the protection of the Δ^5−6^ alkene as an epoxide [47]. However, neither a complete BS biosynthetic pathway nor a total chemical synthesis for BS has been reported yet. Therefore, we propose a further study to understand the underlying mechanism of BS biosynthesis in different plants as well as an economic and efficient chemical synthesis process. The former will serve the purpose of finding the role of cytosolic and plastid BS in plants and may also produce clues for the economic and efficient chemical synthesis of BS.

## 5. Systemic Effect

### 5.1. Central Nervous System

BS containing plants show antinociceptive [48], anxiolytic, and sedative effects [49] in rats, but such findings in humans are not available. Neither the brain region nor the pathway affected by BS has been studied extensively yet. It has been shown that the effect of BS is somewhat similar to diazepam but whether the mechanism of action is similar or not has not been studied [50]. It has been proposed that BS is effectual by interacting with GABAA receptor, but there is no confirmatory evidence for this claim [49]. BS has been shown to potentiate the binding of other compounds to muscarinic receptors [51]. However, whether or not BS binds to the muscarinic receptor itself is not known. Studies in immortalized mouse hippocampal cell line HT22 showed that BS prevents oxidative damage and neurotoxicity [52] and a series of other studies showed the beneficial effect in preventing neuronal damage [53,54]. There is evidence that BS crosses the blood brain barrier (BBB), but fundamental studies regarding the efficacy and efficiency of BS to cross BBB have not been undertaken. A comparative study has been done with other phytosterols like campesterol and sitosterol to check the efficiency in passing the brain endothelial monolayer where the reason behind the irreversible passage of the plant sterols across the endothelial monolayer was found to be the molecular complexity of the sterol side chain. A possible explanation for the difference of phytosterols in passing the BBB may be the different esterification rate within the endothelial cells [26]. Recent studies have shown that BS alone [55] or as extract [56] increases neural stem cell proliferation. However, further studies are recommended for potential applications in tissue engineering.

### 5.2. Skin

According to the Norwegian Food Safety Authority (Mattilsynet, NFSA, Oslo, Norway), BS has a skin conditioning effect and is used in sunscreen, moisturizer, body wash, and anti-aging cosmetic preparations (NFSA, 2012). Skin is one of the paths of BS excretion. It has been reported that BS inhibits the production and mRNA expression of thymic stromal lymphopoietin through blocking of caspase-1 and nuclear factor-kB (NkB) signal pathways in the stimulated human mast cell line, HMC-1 cells. Even though this study showed the potential therapeutic effect against atopic dermatitis, studies on long-term use of BS on the skin need to be conducted [57].

### 5.3. Cardiovascular System

BS has beneficial effects on the cardiovascular system and prevents different cardiovascular diseases except for patients with ABCG5 and ABCG8 mutation [11,58]. However, there is no study regarding its effects on cells within the heart: the cardiomyocytes and the cardiac pacemaker cells. Although some studies point to the possibility that elevated plasma phytosterol concentrations could contribute to the development of premature coronary artery diseases, extensive safety evaluation studies have been conducted for these compounds, and they are considered safe [59].

### 5.4. Liver

BS containing diets change the live ultra-structure and such differences are observed in both young and adult mice fed with BS [60]. Pathophysiology of the liver is also affected by BS. For example, BS prevents gallstone formation and decreases serum and liver cholesterol [61], but such preventive effects are observed only at high doses [62]. The effect of BS on different metabolizing enzymes has not been studied and therefore sufficient information regarding the metabolism of drugs that are affected by BS is not available.

### 5.5. Endocrine System

BS possesses a weakly estrogenic effect and alone or in combination with progesterone, it inhibits the expression of intercellular adhesion molecule-1 [63] and testosterone propionate induced prostate hyperplasia [64] as well as reducing pregnenolone production [65]. Even though the molecular effect of BS on the tonicity of the uterus has been studied [66], the long-term effect of BS on different hormones has not been studied and therefore further study is required.

### 5.6. Reproductive System

The effect of BS on the reproductive system is contradictory. Study on American mink shows increased male fertility due to BS intake [67], but other studies in male rats [68] and goats [69] show the opposite effect on reproduction. The level of sex hormones such as testosterone in males and estradiol in females is increased due to BS intake in rats [70]. Whether or not this increase has any clinical significance has not yet been studied.

### 5.7. Wound Healing

Different plants containing phytosterols like *Mimosa tenuiflora* have been used for decades as a remedy in the treatment of wounds and burns of the skin. This can be explained by the re-epithelialization process in wounded areas which is believed to be aided by BS. So, the ability to heal, together with the anti-inflammatory and antimicrobial activity of BS demonstrate its potential in tissue engineering applications [71].

## 6. Pharmacokinetic Studies

**Figure 7:**
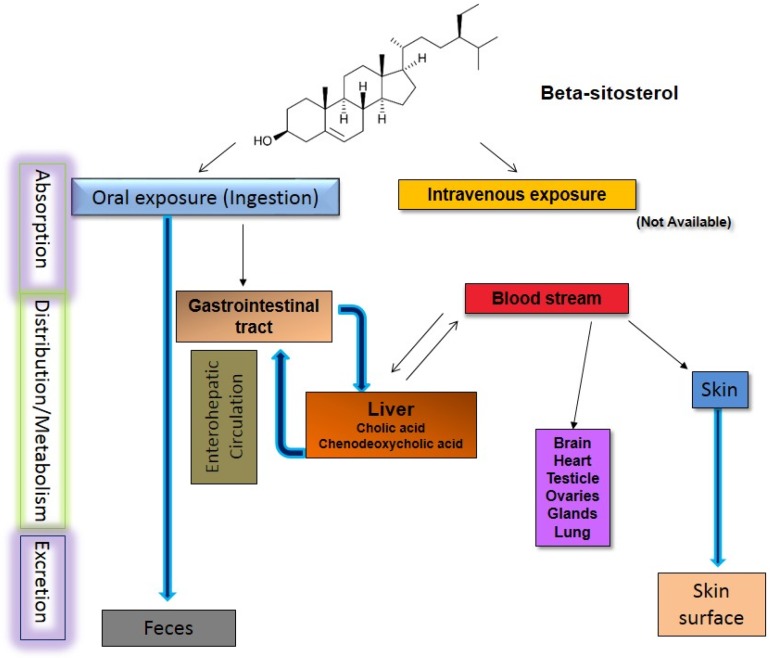
Pharmacokinetics of beta-sitosterol.

Pharmacokinetics and the bioavailability of BS (Figure 7) have been reported both in animal [72,73] and human [74,75], alone as well as with other compounds [76], including sex hormones [77] and cholesterol [78] mostly. Reports in diseased humans have also been reported [79]. Even though the metabolism of BS was described 45 years ago [80], the detailed metabolic turnover, absolute oral bioavailability, clearance, and volume of distribution for BS measured in healthy subjects have been reported only recently [75]. Generally, it has been considered that BS interrupts the recirculation of bile acids and/or reduces the absorption of cholesterol in the gut [81,82,83,84,85]. However, substantial experimental evidence is needed to propose the primary molecular mechanism about the physicochemical competition between cholesterol and BS and other phytosterols for micellar incorporation and uptake at the gut lumen [86].

Structurally cholesterol and BS are different from each other only by the additional ethyl group at the C-24 position in the latter. The absorption of BS is one-fifth of that of cholesterol [87]. Field and Mathur (1983) attributed the inadequate esterification of BS to poor absorption, but there is no experimental evidence to this claim [88]. BS absorption is higher in females than males [89], but no satisfactory explanation has been provided until now. The study shows that efflux transporters play a role in absorption of BS and it has been shown that loci on chromosomes 14 and 2 in rats play a role in the concentration of BS but further study is not available [90]. BS is distributed in the adrenal glands, ovaries, brain, testicles, as well as skin [75,89,91]. BS is metabolized to different compounds in the liver and other tissues forming different compounds. BS is converted to autoxidation products in the GI tract as well as before excretion via feces [92]. A general study regarding hepatic enzyme metabolism is available [93,94] but until now there has not been any study regarding the differential roles of different hepatic enzymes in its metabolism even though the role of BS has been studied while studying the effect of different hepatic enzymes on other drugs [95]. Bacterial conversion products from BS that have easily been identified and measured in the gut are 24-ethyl-coprostanol, as the most prominent component, as well as coprostanol, and 24-ethyl-coprostanone as minor components [96]. About 80% of the absorbed BS is excreted via feces [80] and the rest is excreted via skin [97]. Generally, excretion via feces is rapid but differential excretion is observed based on the disease condition of the colon [98] and physical stress [99]. Coeliac disease has been attributed to impaired BS absorption [100] but whether or not the presence of higher BS in the colon has a causal effect on coeliac disease needs to be studied.

## 7. Therapeutic Potentials

### 7.1. Antioxidant Activity

Several findings suggest that BS has antioxidant property [5,101]. It has also been shown to modulate antioxidant enzymes and human estrogen receptor [92]. It has been reported from a study that BS reduced Oxygen free radical and Hydrogen Peroxide levels in Phorbol myristate acetate (PMA) stimulated RAW 264.7 cells but does not function as a radical scavenger [102]. Glutathione peroxidase (GSH) and Mn superoxide dismutase (SOD) activities are decreased significantly by BS treatment [103]. BS does not affect Cu-Zn SOD activity, but whether BS promotes up-regulation of Mn-SOD needs further investigation.

### 7.2. Angiogenic Effect

BS plays a role in blood vessel formation and thus possesses potentials in wound healing [104]. However, there has been no experimental study on the mechanism of wound healing until now. Choi et al. (2002) shows blood vessel formation in ischemia, but further study regarding the feasibility of using BS as a therapeutic agent for ischemic stroke has not been conducted [105].

### 7.3. Antihyperlipidemic and Anti-Atherosclerosis Effects

BS is recommended for the prevention of different cardiovascular diseases [106,107,108] and the FDA has approved BS for the treatment of hyperlipidemia [109]. It prevents the absorption of cholesterol by displacing it from micelles [110] and thereby decreasing the amount in plasma [83,84,111,112,113,114]. In combination with other statins, it increases the potency of those statins [115]. Still further studies are required to resolve existing debate regarding its role in treating hypercholesterolaemia [116]. Study regarding the role of BS on the upregulation of paraoxonase-2 needs to be conducted in order to substantiate the claim made by Rosenblat et al. (2013) regarding the beneficial effect of simvastatin in combination with BS [117]. BS has also been related to sitosterolemia but not as a causal agent for the development of coronary heart disease in sitosterolemic patients [118]. Further experimental evidence is required.

### 7.4. Antipyretic Activity

A study on rats has shown that the antipyretic effect of BS is comparable to that of aspirin [119]. The preparations and extracts of plants containing BS have also been shown to have antipyretic activity [14,120]. This effect is comparable to that of the standard antipyretic drug, aspirin, but the detailed mechanism has not yet been studied.

### 7.5. Anti-Inflammatory Activity

BS possesses anti-inflammatory activity in human aortic cells [121] as well as in rats [122,123]. Several studies in animals have indicated that BS reduces the secretion of pro-inflammatory cytokines, TNF-α as well as edema [119,124,125] and increases anti-inflammatory cytokines [126]. Chronic treatment with BS reduces its anti-inflammatory potential [127] and it does not affect the mast cells in histamine release [128] and the arachidonic acid pathway [129]. Whether BS inhibits either myeloperoxidase or adenosine deaminase activity or both needs further investigation. Again, whether BS inhibits or not IL-1β and TNF-α levels by increasing calcium uptake in activated neutrophils in a concentration- and time-dependent manner through l-type voltage-dependent calcium channels, phosphoinositide kinase-3, intracellular calcium and microtubule modulation, and thus promotes the anti-inflammatory activity as proposed by Liz et al. (2013) [130], requires experimental evidence. Even though Mahanjan and Mehta (2011) have shown therapeutic potential in allergic asthma by a chronic study in pigs it has not been used for clinical trials in humans [131].

### 7.6. Anti-Arthritic Activity

It has been reported from a study that the plant extract containing BS has significant anti-arthritic activity [132]. According to Moreau et al. (2002), BS decreases the activation of NF-κB transcription factor in PMA-stimulated macrophage cells [133]. However, further investigations are required regarding the therapeutic potential of BS to treat arthritis.

### 7.7. Immune Modulation and Anti-HIV Effect

BS has been shown to act as a powerful immune modulator [134]. BS exhibits immune-modulating activities in HIV-infected patients [135]. It has also been reported that BS targets specific T-helper (Th) lymphocytes, increasing Th1 activity and improving T-lymphocyte and natural killer (NK) cell activity [135,136]. In another study it was observed that BS maintains stable CD 4 cell counts in AIDS, declines apoptosis of CD 4 lymphocytes slightly, thereby slowing HIV. A significant decrease in IL-6 levels in the same study leads to a further claim that there is slowing down of viral replication rates in infected cells thereby decreasing viral load [137]. Neurath et al. (2005) proposes BS as an envelope virus neutralizing compound (EVNC) and thus acting as an HIV-1 entry inhibitor [138]. This claim has been substantiated by the fact that the EVNCs in the body fluid neutralize viruses in the blood stream and elicit an immune response to the neutralized authentically folded virus particle [139,140]. Even though the effect of BS on entry and exit out of the cell is not available, it is evident that BS facilitates the development of a potentially protective immunity against HIV. However, further study for considering BS as potential therapeutic agent has not progressed. Therefore, extensive study is suggested.

### 7.8. Anti-Cancer Effect

Experimental and epidemiological studies have shown the efficacy of BS in treating different types of cancer via different pathways. One recent review documented in detail regarding this [141]. However, most studies have been carried out with different cancer cell lines, where different cellular factors are affected by BS, but ultimately cell lines undergo apoptosis. For breast cancer, MDA-MB-231 [142], U937 [143], HL60 [144], MCF-7 [145]; for colon cancer, HT-29 [146,147], HT116 [105], COLO 320 DM [148], Caco 2 [149]; for prostate cancer, LNCaP [142], PC-3 [150], 22Rv1 [151], DU145 [151]; for fibrosarcoma MCA-102 [152]; for uterine cervix cancer, SiHa cells [153]; for larynx carcinoma, Heps [154] have been studied. Studies on the antitumor effect of BS in animals are relatively few. For colon carcinogenesis, studies were done on rodents and on rat prostate [155,156]. For the former, the result is positive, but for the later the result is negative. These studies with BS are extensive and explain the anticancer mechanism of action. For example, several studies have indicated that BS inhibits the growth of various cultured cancer cell lines that are associated with the activation of the sphingomyelin cycle [147,157,158]; cell cycle arrest [150,159], and the stimulation of apoptotic cell death [105,160]. BS isolated from various plants promotes apoptosis by increasing first apoptosis signal (Fas) levels and caspase-8 activity [8], phosphorylation of extracellular signal-regulating kinase (ERK) and p38 mitogen-activated protein kinase (MAPK) [161], inhibition of cancer cell proliferation, even at low concentrations with no cytotoxic effect on noncancerous cells [152], modulation of antioxidant enzyme levels in pathogenesis [103], arresting of cells at G2/M phase in cancer cells [150], and decreasing free radical generation in vitro [102,162]. BS induces a reduction in membrane sphingomyelin and an increase in the ceramide levels in some tumor cells [147,157]. The efficiency of ceramide playing a role in the activation of the extrinsic pathway as suggested by observations of death receptor clustering in ceramide-rich lipid rafts has not been studied for experimental evidence [163,164]. In addition to the negative effect of BS on cell growth, BS treatment of tumor cells is associated with increased apoptosis [165]. Even with these extensive studies, there is still very little translational research for treating different cancers. One possible explanation could be its lower efficacy and another could be fewer chances of patents by pharmaceutical research organizations. Therefore, research in an academic setting is needed.

### 7.9. Anti-Diabetic Effect

Oral treatment with BS increases the fasting plasma insulin levels. There is a corresponding decrease in fasting glycemia when BS is administered orally. In addition, it improves the oral glucose tolerance test with an increase in glucose-induced insulin secretion [166]. These effects are comparable to that of the standard anti-hyperglycemic drug Glibenclamide. However, the hypoglycemic effect manifested by BS by increasing circulating insulin levels and the mechanism of this increase need further study. A study has shown that treatment of diabetic rats with BS prevents the development of diabetes as well as ameliorating diabetic complications along with other diseases such as arthritis [101]. The same study showed that BS increases glucose uptake in adipocytes and stimulates adipogenesis in differentiating preadipocytes. Paradoxically, it also induces lipolysis in adipocytes which have not been attenuated by insulin and co-incubation with epinephrine. Like insulin, it down-regulates GLUT4 gene expression, but a confirmatory study is required to ensure that elevation of glucose uptake by BS in adipocytes is unrelated to the de novo synthesis of GLUT4 and whether lipolysis is associated with down-regulation of Akt and PI3K genes. Even though due to the unique effects of BS on the regulation of glucose uptake, adipogenesis, and lipolysis in adipocytes supports its potential to be utilized in diabetes and weight management [167], no clinical study has yet progressed. Furthermore, a study should be conducted on whether or not BS has any role in insulin sensitivity and glucagon secretion.

### 7.10. Anti-Pulmonary Tuberculosis Effect

According to the double-blind, randomized, placebo-controlled trial conducted by Donald et al. (1997) on culture-positive pulmonary tuberculosis patients, BS has been found to have a significant improvement in weight that is lost due to pulmonary tuberculosis [168]. The same study showed that patients receiving BS exhibit notable differences in certain hematological parameters, including increased lymphocyte, eosinophil, and monocyte counts. The detailed mechanism of this effect has not yet been studied. The efficiency of BS as immune modulating agent in case of multi-drug-resistant tuberculosis needs further investigation.

### 7.11. Antimicrobial Activity

BS obtained from different plants shows antibacterial and antifungal activity without toxicity in brine shrimp lethality assay [169,170,171]. The formulation or plant extract containing BS shows mosquito larvicidal activities [172] and antitrypanosomal activities [173]. BS has been reported to have antibacterial activity with a comparable zone of inhibition to other standard antimicrobial agents [32,174]. The prime limitation of these studies is the inadequacy in explaining the mechanism of actions. Kanokmedhakul et al. (2005) attributed this to the ability of BS to inhibit bacterial cell surface protein, “sortase” thus preventing transpeptidation [175]. Betasitosterol-3-*O*-glucopyranoside (BSG), a derivative of BS, inhibits bacterial cell adhesion to a fibronectin indicating that modification of BS is needed to exert its antibacterial effect [176]. However, no study has been conducted regarding the mechanism of anti-protozoal, anti-larvicidal or anti-fungal effects. Again, no study has been run to ensure any effect of BS on the ribosome, RNA transcription, DNA replication or the enzymes involved in central dogma. A detailed study is proposed with a hope of obtaining a good alternative to the antimicrobial agent in this current era of antimicrobial resistance.

### 7.12. Miscellaneous

BS has been reported to have anthelmintic properties alone [177] and in combination with one of its derivatives [178]. The mechanism is not well defined and no study has been conducted yet on this. Various plant extracts containing BS can neutralize different snake venoms [179]. However, the mechanism has not yet been discovered with experimental evidence. BS also has a significant role in the treatment of androgenic alopecia [180,181] and studies with human clinical trials have shown positive results [182]. There are some marketed preparations with BS claiming its efficacy in this case, but long term safety data is not available. There are also some marketed preparations that claim beneficial effects in benign prostate hyperplasia (BPH) [183,184,185] and on lower urinary tract infection [186]. However, the molecular mechanism of any of these claims has not yet been established. Lomenick et al. (2015) discovered some protein receptors of BS, but more research in required [187].

## 8. Toxicity

Even though the United States National Toxicology Program (NTP) reviewed toxicological effects of BS about 18 years ago (NTP, 1997), many study results need to be re-evaluated based on the latest findings. A high level of BS concentrations in blood has also been correlated with increased severity of heart disease in men who have previously been suffering from heart attacks [188]. There are drug interactions of BS with Ezetimibe and atorvastatin, pravastatin, simvastatin, and lovastatin or fluvastatin [189,190]. Ezetimibe inhibits the uptake of BS which provides the molecular basis for the treatment of sitosterolemia with ezetimibe [191]. Short-term repeated administration of BS in rats has been reported not to produce gross or microscopic lesions in liver or kidney [68] but such a report on humans taking BS for a long time has not been produced. An extensive toxicological study has shown high LD_50_ in rats (>2 gm/kg) [5]. According to JECFA (2009), acceptable daily intake (ADI) is 40 mg/kg∙BW/day; No-Observed-Adverse-Effect-Level (NOAEL) is 4200 mg/kg∙BW/day; Margin of Safety (MOS) is 210 mg/kg∙BW/day and 8.3 mg/kg∙BW/day for systemic and cosmetic products respectively. These values are calculated approximately from phytosterol mixtures, not directly from BS solely and therefore values based on BS are highly recommended. BS inhibits mutagenicity [177], prevents chromosomal breaks [192], and shows no genotoxic effects [193]. Even though one study found its potentially harmful effect on the reproductive system [194], later study found that it does not have an effect on the reproductive system [195]. However, there is no study regarding the long-term effect of BS in the human reproductive system. In a recent study, it was shown that high exposure of BS is related to impaired hepatic and intestinal ATP-binding cassette transporters G5/8 and possesses potential risks of blood-brain barrier integrity in diabetic rats [196]. Another main limitation of BS toxicity study is the unavailability of its readily oxidized products.

## 9. Drug Delivery with Beta-Sitosterol

Side chain double bonds increased sterol mobility considerably in HPLC, which reflected decreased hydrophobicity of the molecule. However, the change in hydrophobicity depended upon the position of the side chain double bond: sterols with double bonds at the C22 position were more hydrophobic than sterols with double bonds at the C24 position. Increases in the side chain length, by the addition of methyl or ethyl groups, resulted in decreased HPLC mobility and increased hydrophobicity, whereas the insertion of a double bond in one or both fatty acyl chains decreased hydrophobicity [197]. BS has poor absorptivity and therefore additives for enhancing its bioavailability or drug delivery with different dosage forms did not progress extensively even though its pharmacokinetic and bioavailability data was reported long ago [72,73,80]. Liposomal BS has been reported along with its ability to increase natural killer cell activity and decrease metastatic colonies in the lungs significantly in comparison to the control group [198]. BS has been reported to act as a model drug or substance for a novel formulation [199] and to test the efficacy in emulsion form [200]. It has also been reported as a formulation additive for stable micellar formulation [201], and for novel bio-active lipid nanocarriers for stabilization and sustained release [202]. It enhances drug release from a gel preparation [203], activity in phyto-vesicle preparation [181], oral absorption efficiency [204] and the sustained release of hormone [205]. More works have been conducted with BSG for enhancing absorption of a different formulation of genes [206,207] and drugs for skin [208]. In combination with another drug, BS has been reported to enhance nasal [209] and intestinal absorption [210], or to deliver the drug to the specifically targeted organ, such as liver [207,211]. However, no clinical trial has been conducted with any of these formulations. Designing intelligent drug delivery for increasing intestinal BS absorption is promising, especially for site-specific therapy of cancer, because of the non-toxic nature of BS to non-cancer cells. Therefore, we propose clinical trials of BS liposomal drug delivery for breast cancer, colon cancer etc.

## 10. Future Research Directions

BS has been reported to have beneficial effects in different diseases, but it has not developed as an independent drug mostly because of its relatively lower efficacy and the development of other drugs with higher efficacy. For example, both BS and glucocorticoid, dexamethasone (DX) have anti-inflammatory effects, but DX has obtained unprecedented approval being a standard drug since its inception [212] even though it lacks sufficient clinical trials [213]. Now research with BS which has fewer side effects might lead to the development of a newer anti-inflammatory drug. New study design should be made on drug delivery to compensate its lower efficacy and poor absorptivity. Over-generalization of systemic pharmacological effects of all phytosterols by regulatory agencies such as EFSA, WHO, FAO and attributing generalized statements on BS is also considered a big challenge. BS is one of the phytosterols which is structurally different from other phytosterols such as campestral, brassicasterol, ergosterol etc. It is highly likely that phytosterols have differences in their effects, at least in their efficacy. EFSA, USFDA, joint FAO/WHO published a report on phytosterols as food supplements or additives without any specific emphasis on any individual compound. This tendency to generalize the effect of phytosterols has limited the study of the individual effect of different phytosterols. This effect is also observed in many clinical trials. However, most of the trials do not categorize phytosterols, but rather administer a mixture of phytosterols. Such oversimplified statements are vague and do not lead to the development of newer therapeutics. However, several clinical trials that have been carried out with BS are multicentric, placebo-controlled, double-blind [183] or simple comparative study [15,186,214] for the treatment of BPH [183,184,185]; lower urinary tract infection [186]; hypercholesteremia [214], immunosuppression and inflammation [135], rheumatoid arthritis [215] and androgenetic alopecia [182]. The results have shown some beneficial effects, but neither long-term safety data nor clinical trials with drug delivery aiming to overcome its lower efficacy are available. This is mostly due to lack of sufficient research-based information on BS needed for such official publication. Even the Norwegian Food Safety Authority (NFSA, 2012) published a risk profile of BS but its directives are mostly based on studies on phytosterols in general, not BS alone. General directives for phytosterols may serve as a guideline for the use of a phytosterol mixture, but it cannot serve the purpose when BS is used and marketed alone. Even though the FDA has approved the manufacturer’s claim of the beneficial effects of BS against coronary heart disease, most of the manufacturers commercialize it to treat alopecia and BPH, even though there is still no long-term convincing result regarding the efficacy of BS against alopecia and BPH. Pharmaceutical research organizations have a relatively low interest in research with BS. Therefore, research in an academic setting as well as through funding from national and international organizations such as FAO, WHO, EFSA etc. to find its long term effects at the molecular and cellular level is recommended. Research for improving efficacy via chemical modifications or via intelligent drug delivery to improve absorptivity and specificity is also recommended. Such research is urgent for at least two reasons: one is caution and another is hope. The caution is regarding its safety for chronic public use, either systemically or topically. The hope is for the pharmaceutical research organizations to set newer avenues to find out modern alternatives to current therapeutic agents. Even though BS has many important roles in different diseases, it has been neglected mostly because of its lower potency in most of these cases. However, the fact is that its relatively higher safety in comparison to other available drugs being used to treat different diseases has been ignored. An extensive risk-benefit study, at least in the academic setting, is therefore highly recommended.

## Figures and Tables

**Table 1:** ^1^H and ^13^C NMR chemical shift values for BS * [35].

Position	^1^H	^13^C
3	3.53 (tdd, 1H, *J* = 4.5, 4.2, 3.8 Hz)	72.0
5	5.36 (t, 1H, *J* = 6.4 Hz)	140.9
18	1.01 (s, 3H)	12.0
19	0.68 (s, 3H)	19.0
21	0.93 (d,3H, *J* = 6.5 Hz)	19.2
26	0.83 (d, 3H, *J* = 6.4 Hz)	20.1
27	0.81 (d, 3H, *J* = 6.4 Hz)	19.6
29	0.84 (t, 3H, *J* = 7.2 Hz)	12.2

* Assignments made on the basis of COSY, HMQC, and HMBC correlations; Chemical shift values are in δ (ppm); Coupling constants are in Hz.
